# Life course approach to health: a paradigm shift to build global health resilience for person-centred healthcare in a turbulent world?

**DOI:** 10.7189/jogh.15.03049

**Published:** 2025-12-12

**Authors:** Zheyi Fang, Jinkou Zhao, Fan Wu

**Affiliations:** 1 School of Public Health, Fudan University, Shanghai, China; 2 Shanghai Institute of Infectious Diseases and Biosecurity, Fudan University, Shanghai, China; 3 Fudan Institute for Advanced Studies in Global Health, Fudan University, Shanghai, China; 4 The Global Fund to Fight AIDS, Tuberculosis and Malaria, Geneva, Switzerland; 5 Shanghai Medical College, Fudan University, Shanghai, China

## Abstract

Intersecting crises, such as complex health threats, deep-rooted inequities, and intergenerational vulnerabilities, expose the critical limitations of fragmented, disease-specific global health programmes. While the life course approach to health (LCAH) is theoretically recognised, it is inadequately implemented in global health governance and financing. In this viewpoint, we aim to reposition LCAH as an operational blueprint for the future global health agenda. Based on a ‘problem-principles-pathway’ logic model, we articulate why and how LCAH can address key challenges of today’s global health context. The LCAH is reframed around three actionable shifts: from vertical programmes to holistic integration via person-centred, primary care-based systems; from opportunity-equality to temporal equity, addressing cumulative disadvantage across the lifespan; and from short-term reactivity to proactive resilience, built through sustained investment in ‘health capital’ at critical life stages. We call for coordinated action between policymakers, donors, global health actors, and community health workers to operationalise this framework. While barriers like short-term political and financial cycles still need be overcome, the LCAH offers a vital paradigm shift for building societal resilience and achieving equitable health trajectories in an uncertain world.

Global health is entering a period of compounding risks and threats. The simultaneous burden of infectious and non-communicable diseases (NCDs), alongside demographic and epidemiological transitions, climate-related shocks, digital disruption, and conflicts, are expanding the traditional boundaries of ‘health’ and ‘health systems’. These dynamics have revealed the inadequacy of fragmented, disease-specific, and reactive interventions and call for a fundamental reimagination of global health strategies [1].

The life course approach to health (LCAH) [2,3], originating from mid-20th-century epidemiological and developmental science and formally advanced in WHO’s 2025 Framework to Implement a Life Course Approach in Practice, offers a new paradigm for tackling these challenges. Although its principles are increasingly embedded in national health strategies, they have yet to be fully translated into global governance, financing, and coordination mechanisms [2].

In this viewpoint, we aim to reposition and operationalise LCAH for the future global health agenda. Following the ‘problem-principles-pathways’ model, we first outline the contemporary global health landscape. Then, we demonstrate how LCAH addresses key challenges, including complexity, inequity, and intergenerational vulnerability. Finally, we suggest how key actors could integrate LCAH into global-level action.

## THE CHANGING GLOBAL HEALTH CONTEXT CALLS FOR LCAH

Today, global health is facing a convergence of persistent and emerging threats. NCDs now account for 74% of global deaths, while infectious diseases continue to cross borders of countries worldwide [4]. Mental health disorders, antimicrobial resistance, and recurrent epidemic outbreaks further stretch systems already operating at their limits [5].

Simultaneously, the boundaries of ‘health’ have expanded beyond biomedical risks to include climate-related shocks, fragile food systems, digital inequalities, and environmental degradation. These interconnected risks have overwhelmed fragile health systems, particularly in low- and middle-income countries (LMICs) [5].

Demographic transitions also highlight significant temporal global health inequities. Many high-income countries are experiencing rapid population ageing, while LMICs face high maternal and child mortality due to underinvestment in early-life health systems. In sub-Saharan Africa, more than 60% of the population is under 25 years of age, while the number of youth is set to rise by 42% by 2030, indicating potential health demand, labour markets, and fiscal pressures [6].

Crucially, these challenges have a cumulative influence across an individual’s life span. Without life-course-oriented action, today’s early-life risks may become tomorrow’s morbidity, productivity loss, and entrenched intergenerational inequity [7].

## A SOLUTION TO FRAGMENTATION, INEQUITY, AND INTERGENERATIONAL VULNERABILITY: REIMAGINING THE DEFINITION OF LCAH

The rise of populism, the collapse of multilateralism, and the withdrawal of donor commitments have likewise raised worries over the sustainability of health financing [8,9]. Notably, the 2025 foreign aid freeze under the Trump Administration disrupted at least USD 900 million in global health funding [10], resulting in closures of clinics, workforce reductions, and service interruptions for vulnerable populations in many LMICs.

The LCAH approach can help ensure sustained commitments and equitable access to health services, as it views health as a cumulative trajectory shaped by biological, social, economic, and environmental exposures from preconception through old age [3,10]. It offers a comprehensive framework by engaging with the cumulative nature of health across the lifespan, structural determinants, and even geopolitical realities, to address fragmentation, reduce inequity, and build health system resilience. As such, it holds relevance as a guiding framework for global health action (**Table 1**).

**Table 1:** Aligning the global health context with the LCAH approach

Global health context		LCAH	
**Health challenges**	**Current global health actions**	**Principles**	**Proposed solutions**
Complexity: emerging and persistent threats are interwoven; expanded boundaries of ‘health’ system fragility	Fragmented, vertical	Health is shaped by multisectoral exposures	Shifting from fragmented vertical programs towards coherence and person-centred systems across the lifespan, with primary healthcare as the entry point
Inequity: deep-rooted disparities in health capital and opportunities	Temporal equity neglected	Disadvantages accumulate over time	Shifting from providing equal services at a single point in time to ensuring equitable health trajectories across a lifetime to address the cumulative disadvantages
Temporality: health challenges accumulate and compound across generations	Lack of programme continuity and long-term investment	Early investment builds health capital	Redefining resilience not as a reactive emergency response, but as the proactive outcome of sustained, long-term investment in ‘health capital’

LCAH – life course approach to health

### Overcoming fragmentation to address the complexity

Despite the growing complexity of health threats, global health responses remain siloed to disease-specific programmes, short-term project cycles, and fragmented delivery platforms. Since the establishment of the United Nations system, and especially the era of the Millennium Development Goals, the proliferation of global health actors has resulted in the mobilisation of an unprecedented amount of resources, but also to the intensification of coordination challenges [11].

To address this fragmentation, the LCAH offers a coherent, system-oriented perspective. It promotes the integration of services across all life stages, linking reproductive, maternal, child, adolescent, adult, and geriatric care within a broader systems approach that includes social protection, education, labour, and environmental policies. The LCAH shifts the health system away from vertical disease-specific programmes toward person-centred, coordinated models. It also emphasises strong primary healthcare platforms as critical entry points; as an example, Ethiopia’s Health Extension Programme established a community-based primary healthcare platform that delivers continuous services across the life course, successfully improving access to essential healthcare services [11,12].

### Advancing both opportunity and temporal equity

Equity has long been a priority of global health actions. However, many efforts continue to operate within an ‘opportunity-equality’ paradigm, focussing on ensuring the same service package at a single time point, but overlooking the cumulation of disadvantages over time. As a result, vertical programmes often operate without coordination, potentially leaving patients in transition untreated. For example, adolescents with HIV are experiencing insufficient support during their transition from paediatric to adult care [13].

Rather than treating health inequities as isolated or contemporary, the LCAH provides a temporal lens to understand how they can improve long-term health outcomes and reinforce structural inequity. For instance, tackling child malnutrition and poor early development requires interventions that go beyond postnatal care to address potential upstream determinants such as maternal undernutrition, low female literacy, and cultural exclusion [14]. Therefore, LCAH calls for early, context-specific interventions to promote health during critical developmental windows.

### Addressing intergenerational vulnerability to build resilience

The current short-term and output-driven global health financing mechanism, vulnerable to political cycles and economic volatility, is insufficient for building resilience. The LCAH approach views resilience as a proactive long-term outcome of investment, rather than a reactive response [15]. Any early investment can be seen as important ‘health capital’ for improving lifelong health outcomes, reducing future healthcare costs, enhancing human capital, and buffering communities against shocks.

While constructing ‘health capital’ early in the life course remains important, it is key to note that it can be developed at any time, especially at each of the critical transition points. By tracing where health capital fails to accumulate, LCAH helps identify vulnerable groups and critical intervention points.

## CALL FOR ACTIONS, OPERATIONALISING LCAH IN GLOBAL HEALTH

The contemporary global health challenges necessitate an unwavering commitment to LCAH. Five key actor groups – policymakers, donors, communities, healthcare workers, and researchers – hold distinct, but interdependent responsibilities in this transition.

### Policymakers

Policymakers must reorient health governance toward long-term, system-wide, and intersectoral strategies grounded in strong primary healthcare. This requires fostering policy environments that support intersectoral coordination. Additionally, longitudinal health metrics and forward-looking targets should be set to reflect life-course priorities and guide system reforms. This includes embedding LCAH principles within the Sustainable Development Goals monitoring systems, and mandating Health in All Policies to align fiscal, environmental, education, and labour coordination with LCAH goals [16].

### Donors

Donors should align financing with the intergenerational nature of health. Performance metrics for global health programmes must shift from short-term outputs to indicators reflecting life-course outcomes and intergenerational equity. Financing must shift from curative services toward strategic investments in primary, secondary, and tertiary prevention. This can be facilitated by innovative financing instruments like prevention bonds, results-based financing, and leveraging funds from development banks. Donors should also promote coherence across parallel initiatives through integrated planning, pooled financing mechanisms, and shared accountability frameworks.

### Communities

Communities play a vital role in translating life-course approaches into context-specific actions. Beyond passive service uptake, they should actively participate in co-designing, implementing, and adapting interventions. By building health literacy, strengthening social support networks, ensuring accountability, and creating local solutions, communities can help foster continuity and resilience across the life course.

### Healthcare workers

To close the gap between LCAH theory and practice, life course principles such as integrative longitudinal care, social determinants of health, and intersectoral collaboration must be embedded in the medical and public health education as core competencies. Clinicians must then translate theory into practice. This dual investment in educating the next generation and empowering the current workforce is essential to building a unified, competent, and resilient health system aligned with the life course approach.

### Researchers

Research is critical for operationalising LCAH through robust evidence. Applying a life course perspective requires longitudinal cohort studies and data systems, birth-to-death surveillance, analytical models, and indicators tailored to life-stage-specific. Investment in social, behavioural, and implementation research is equally critical, particularly in LMICs.

## APPENDING QUESTIONS AND OBSTACLES TO THE LCAH OPERATIONALISATION

There are significant barriers to translating the LCAH principle to practice. A primary challenge is the structural misalignment between the long-term, intergenerational nature of LCAH and the prevailing short-term incentives of current global health programmes. This mismatch is compounded by fragmented financing mechanisms and the influence of commercial determinants of health. Another critical challenge is adopting LCAH in resource-constrained settings.

A pivotal opportunity lies in aligning the strategic transitions of major global health initiatives, such as the President's Emergency Plan for AIDS Relief in the USA, Gavi, the Vaccine Alliance, and the Global Fund to fight AIDS, Tuberculosis and Malaria, with LCAH principles. These entities can evolve from vertical models into coordinators of integrated, life-course-oriented systems.

Investing strategically in health across the entire life course is not merely sound medicine; it is the cornerstone of societal resilience, economic productivity, and human dignity in an increasingly uncertain world. An LCAH blueprint is essential for global health programmes in navigating the turbulence ahead.
